# Purification, characterization and procoagulant activity of polysaccharides from *Angelica dahurice* roots

**DOI:** 10.1186/s13065-017-0243-y

**Published:** 2017-02-10

**Authors:** Jinmei Wang, Pengli Lian, Qi Yu, Jinfeng Wei, Wen-yi Kang

**Affiliations:** 1 Institute of Chinese Materia Medica, Henan University, Kaifeng, 475004 China; 2 Kaifeng Key Laboratory of Functional Components in Health Food, Kaifeng, 475004 China

**Keywords:** *Angelicae dahuricae Radix*, Polysaccharides, Procoagulant

## Abstract

Five polysaccharides, namely ADPs-1a, ADPs-1b, ADPs-2, ADPs-3a and ADPs-3b, were extracted from *Angelicae dahuricae Radix*, purified, and identified by high performance gel permeation chromatography (HPSEC), gas chromatography (GC), Fourier transform infrared (FT-IR) spectrometer and nuclear magnetic resonance spectra (NMR), including the determination of procoagulant activity in vitro. The average molecular weight (*Mw*) of the polysaccharides was 153,800, 8312, 111,700, 3766 and 96,680 g/mol, respectively. Coagulation assays indicated that ADPs-1b, ADPs-2, ADPs-3a and ADPs-3b had procoagulant activities. ADPs-1b exerted the procoagulant activities through intrinsic pathway, extrinsic pathway and increased the content of FIB in vitro. ADPs-2 exerted the procoagulant activities through intrinsic pathway and extrinsic pathway. ADPs-3a had procoagulant activities and the activity was associated with the intrinsic pathway and increased the content of FIB. ADPs-3b exerted the activities through extrinsic pathway and increased the content of FIB.

## Background

*Angelicae dahuricae Radix*, named ‘Baizhi’ in Chinese, has been a well-known traditional dietary and medicinal plant for several 1000 years. It has traditionally been used for treatment of headache caused by the common cold, asthma, coryza, hypertension, vitiligo, psoriasis and photodynamic therapy. Pharmacological research showed that *A. dahuricae Radix* had antioxidant [1], antibacterial [2], anti-tumor [3] and analgesic [4] activities. The bioactive components mainly contained coumarins [5], volatile oils [6], polysaccharides [7] and trace elements [8]. Coumarins had been intensively studied in the literatures, and polysaccharides were rarely studied. However. Polysaccharides have many biological activities. It has been reported that polysaccharides from *A. dahuricae Radix* had antioxidant activity [9], can promote the proliferation of rat skin cells cultures in vitro [10] and enhance the ability of F81 cells to resist canine parvovirus infection [11].

The literature search showed the water extracts from *A. dahuricae Radix* had obvious hemostatic effect [12]. Till date there is no investigation reported on the bioactive components of water extract from *A. dahuricae Radix* for hemostatic effects. In this paper, water-soluble polysaccharides were extracted and purified, and its procoagulant activity in vitro was studied which could provide a evidence for clinical application of polysaccharides from *A. dahuricae Radix*.

## Methods

### Plant material

*Angelicae dahuricae Radix* were purchased in April 2013 from the golden pieces of Chinese Medicine Co., Ltd. of Yuzhou (Henan, China) and were identified by Prof. Chang-qin Li. The voucher specimens were deposited at traditional Chinese medicine research Institute of Henan University.

### Animals

Male rabbit (2.0–2.5 kg), was obtained from the Experimental Animal Center of Henan Province (Zhengzhou, Henan, China, No: 14-3-7). It was maintained under a 12/12 h light/dark cycle, at 25 ± 2 °C and humidity 45–65%, with free access of food and water. The animal procedures were approved by the ethical committee in accordance with ‘Institute ethical committee guidelines’ for Animal Experimentation and Care. Animals were housed in standard cage.

### Reagents

DEAE-cellulose-52 (Whatman, Germany); Sephadex G-100 (Pharmacia, America); TGL-16 high speed centrifuge (Zhongda instrument factory, Jintan, China); HF6000 Semi-Automated Coagulation Analyzer (Chinese Prescription Medical Instrument Co., Ltd, Jinan, China); LRH-150 incubator (Shanghai Yiheng Technology Co. Ltd., China); stopwatch timer; vitamin k_1_ injection, 2.775 g/L calcium chloride solution, (Tianjin Pharmaceutical Group Co., Ltd. Xinzheng, 1109051); APTT (Lot: 112163), PT (Lot: 105227), TT (Lot: 121116), FIB (Lot: 132058) assay kits (Shanghai sun biotech Co., Ltd.).

### Extraction and purification of polysaccharide

*Angelicae dahuricae Radix* (100 g) were grounded into powder, then extracted three times with 70% ethanol and filtered. Subsequently, the dried power was dipped into 20 volumes of distilled water at 80 °C every 3 h for three times. The aqueous extract was filtered and the supernatant was treated with 95% ethanol (final concentration 70%) at 4 °C overnight, and centrifuged at 10,000 rpm for 10 min. The precipitation was added with Sevage reagent (chloroform/1-butanol, 1:4 v/v) for deproteinization. The crude polysaccharide was obtained through precipitation with 95% ethanol (final concentration 70%) and centrifuged. Then the precipitation was redissolved in water and dialyzed against distilled water for 2 days. Finally, the aqueous extract was lyophilized in vacuum to give the crude polysaccharide (5.11 g).

The crude polysaccharides 300 mg was dissolved in 10 mL distilled water, filtered through 0.45 μm filters and then fractioned by DEAE-52 column (2.5 × 60 cm). The column was eluted with distilled water at 0.8 mL/min, followed by 0.05 M NaCl and 0.1 M NaCl, respectively. The fractions were collected using an automated step-by-step fraction collector and guided for total carbohydrate using the phenol–sulfuric acid method. Three main fractions were collected, dialyzed, lyophilized. These polysaccharides were further purified through a column of Sephadex G-100 (1.5 × 100 cm) and eluted with water at 0.5 mL/min. The purified fraction was combined, concentrated and lyophilized for further study.

### Structural analysis

#### Molecular weight analysis

The molecular weight of polysaccharides was identified by high performance size-exclusion chromatography (HPSEC) in Beijing center for physical and chemical analysis.

#### Monosaccharide composition analysis

Polysaccharide samples (10 mg) were hydrolyzed in ampoules with 2 M trifluoroacetic acid (2 mL) for 3 h at 110 °C, evaporated and added with methanol to remove TFA. Then the hydrolyzates were mixed with 10 mg hydroxylamine hydrochloride and 0.5 mL pyridine and incubated at 90 °C for 30 min. Acetic anhydride (0.5 mL) was added and incubated at 90 °C for 30 min. The mixtures were cooled to room temperature, and filtered through 0.22 μm filters. The resulting alditol acetates were analyzed by GC, which was performed on a Thermo TRACE1300 instrument fitted with FID (280 °C) and equipped with HP-5 column (30 m × 0.25 mm × 0.25 μm). The column temperature was maintained at 110 °C for 5 min, and increased to 190 °C for 4 min at a rate of 5 °C/min, then increased to 210 °C for 10 min at a rate of 3 °C/min. The standard monosaccharides (arabinose, xylose, mannose, glucose and galactose) were prepared and subjected to GC analysis separately in the same way.

#### FT-IR spectral analysis

Polysaccharides were grounded with KBr power, pressed into pellets and then detected in the frequency range of 4000–50/cm.

#### NMR spectral analysis

The NMR spectra of ADP-1a, ADP-1b, ADP-2, ADP-3a, and ADP-3b were obtained by an Avance-600 NMR spectrometer (Bruker Inc., Rheinstetten, Germany). All compounds were dissolved in D_2_O. The ^1^H NMR spectra of ADP-1a, ADP-1b, ADP-2, ADP-3a, and ADP-3b were recorded, ^13^C NMR spectra, the 2D NMR spectra including heteronuclear multiple-quantum coherence (HMQC) and heteronuclear multiple bond correlation (HMBC) of ADP-1a and ADP-2 were recorded.

#### Scanning electron microscope analysis

Polysaccharide samples were fixed on the sample stage, subsequently coated with a layer of gold, and then scanned by scanning electron microscope.

### Anticoagulation time test

Anticoagulation activities of APTT, PT, TT and FIB were analyzed in vitro and the assay was conducted by using rabbit blood collected from rabbit ear vein in the plastic tubes containing 3.8% sodium citrate (citrate/blood: 1/9, v/v). Then, the blood was centrifuged at 3000 rpm for 15 min at 5 °C to obtain the serums. For APTT assay, 25 μL of tested samples were mixed with 50 μL of citrated normal rabbit serum, and then APTT assay reagent was added. Following the mixture were incubated at 37 °C for 5 min. Then 25 mM CaCl_2_ solution (100 μL) was added into the incubated mixture to initiate the reaction. Finally the clotting time was recorded. For PT assay, samples (25 μL) were mixed with serum (25 μL) and incubated at 37 °C for 3 min. While, PT assay reagent (50 μL), which has been hatched for 10 min at 37 °C, was then added and clotting time was recorded. TT and FIB assays were performed according to the manufacture’s specifications. For all clotting assays, blank solvent was used as blank control group, and breviscapine and Vitamin K_1_ were used as positive control group and the time for clot formation was recorded by Semi-Automated Coagulation Analyzer.

### Statistical analysis

All experimental results were expressed as mean ± standard deviation (SD). Statistical analysis was performed with the SPSS 19.0 software. Comparison between any two groups was evaluated using one-way analysis of variance (ANOVA).

## Results and discussion

### Extraction and purification of polysaccharide

**Fig. 1:**
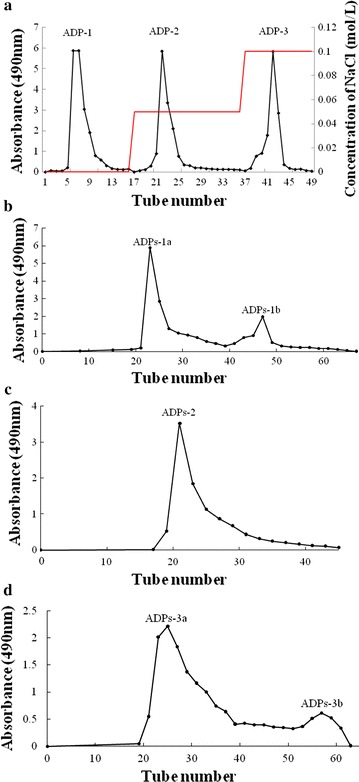
Elution curve of the crude polysaccharides on DEAE-52 (**a**), elution curve of ADP-1 on Sephadex G-100 column (**b**), elution curve of ADP-2 on Sephadex G-100 column (**c**), elution curve of ADP-3 on Sephadex G-100 column (**d**)

Crude polysaccharides (300 mg) were successfully isolated by a series of experimental procedures such as water extraction, deproteination, dialysis, ethanol precipitation and lyophilization. The crude polysaccharides were then separated by using DEAE-cellulose-52 column. Three purified polysaccharide fractions were obtained, named ADP-1 (91.2 mg), ADP-2 (36.5 mg) and ADP-3 (73.6 mg) (Fig. 1a), respectively. Three fractions were further purified by Sephadex G-100. As a result, ADP-1 generated two purified fraction, named as ADPs-1a (30 mg) and ADPs-1b (32.4 mg) (Fig. 1b). ADP-2 generated one purified fraction, named as ADPs-2 (28 mg) (Fig. 1c). ADP-3 generated two purified fraction, named as ADPs-3a (36.2 mg) (Fig. 1d) and ADPs-3b (21 mg) (Fig. 1d).

### Molecular weight analysis

Molecular weight of polysaccharide was a statistical average, which was a representative of similar polymer chain length distributed on average. Generally, the dispersion coefficient (*Mw*/*Mn*) was used to be a judgment whether the molecular weight distributed uniformly or not. As it is shown in Table 1, average molecular weight (*Mw*) of the polysaccharides was 1.538 × 10^5^, 8.312 × 10^3^, 1.117 × 10^5^, 3.766 × 10^3^ and 9.668 × 10^4^ g/mol, respectively.

**Table 1:** Molecular weight of polysaccharides form *Angelicae dahuricae Radix*

Samples	Molecular weight (g/mol)				
	*Mn*	*Mp*	*Mw*	*Mz*	*Mw*/*Mn*
ADPs-1a	1.03 × 10^5^	9.541 × 10^4^	1.538 × 10^5^	2.896 × 10^5^	1.493
ADPs-1b	7.942 × 10^3^	8.187 × 10^3^	8.312 × 10^3^	8.872 × 10^3^	1.047
ADPs-2	8.076 × 10^4^	7.794 × 10^4^	1.117 × 10^5^	1.832 × 10^5^	1.384
ADPs-3a	3.518 × 10^3^	3.551 × 10^3^	3.766 × 10^3^	4.327 × 10^3^	1.070
ADPs-3b	6.751 × 10^4^	8.017 × 10^4^	9.668 × 10^4^	1.815 × 10^5^	1.432

### GC analysis

**Fig. 2:**
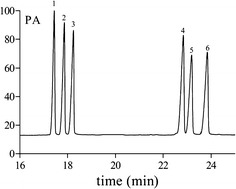
GC spectrum of monosaccharide reference

**Fig. 3:**
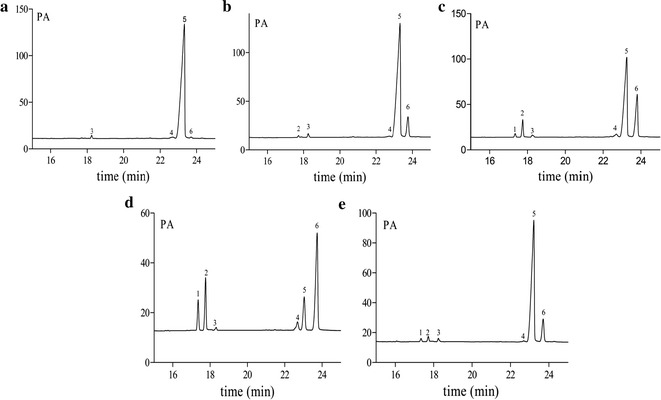
GC spectrum of monosaccharide composition of ADPs-1a (**a**), ADPs-1b (**b**), ADPs-2 (**c**), ADPs-3a (**d**) and ADPs-3b (**e**)

Monosaccharide composition was analyzed by gas chromatography. Based on retention times and content based on every monosaccharide by authentic standards (Figs. 2, 3), the monosaccharide composition of ADPs-1a was xylose, mannose, glucose and galactose in a molar ratio of 0.31:0.22:26.1:0.11. ADPs-1b was composed of arabinose, xylose, mannose, glucose and galactose with a molar ratio of 0.10:0.26:0.07:15.3:1.37. The monosaccharide compositions of ADPs-2, ADPs-3a and ADPs-3b were rhamnose, arabinose, xylose, mannose, glucose and galactose. ADPs-2 was in a ratio of 0.34:1.79:0.35:0.40:15.8:5.59. ADPs-3a was in a ratio of 1.06:2.01:0.13:0.41:1.68:4.97 and ADPs-3b was in a ratio of 0.18:0.36:0.25:0.09:13.5:1.59. According to the literature [13], monosaccharide constituents of ADP were rhamnose, arabinose, xylose, mannose, glucose and galactose, thus differed from our reports and these difference might be related to the source of the *A. dahuricae Radix,* extraction and purification methods.

### Ft-ir

**Fig. 4:**
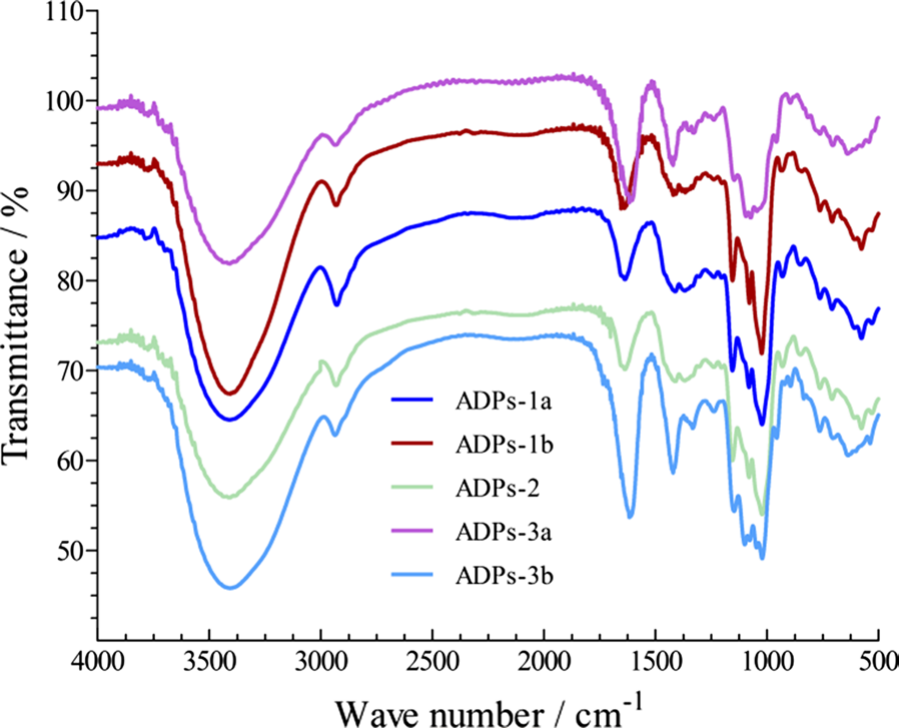
Infrared spectra of polysaccharides form *A. dahurica*

The FT-IR spectroscopy of ADPs-1a, ADPs-1b, ADPs-2, ADPs-3a and ADPs-3b were scanned between 4000 and 500/cm and the results showed the four polysaccharides were similar to each other. As shown in Fig. 4, the absorption band was found in all samples between 3321 and 3375/cm, indicating the presence of hydroxyl group. The appearance of the peaks within the range of 2772–2922/cm was due to the presence of the C–H stretching vibration. The signals at around 1592–1625/cm and 1424–1440 were showing the presence of carboxyl groups. Absorption at 1010–1125 the C–O and C–C stretching vibrations of pyranose ring.

### NMR spectral analysis

**Fig. 5:**
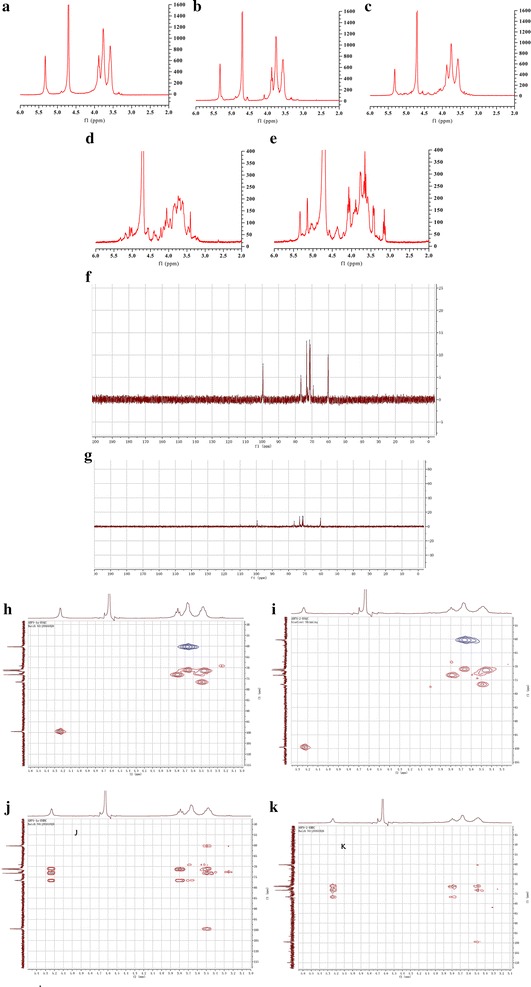
^1^H NMR spectrum of ADPs-1a (**a**), ADPs-1b (**b**), ADPs-2 (**c**), ADPs-3a (**d**), ADPs-3b (**e**), ^13^C NMR spectrum of ADPs-1a (**f**), ^13^C NMR spectrum of ADPs-2 (**g**), HMQC spectrum of ADPs-1a (**h**) and HMQC spectrum of ADPs-2 (**i**), HMBC spectrum of ADP-1a (**j**) and HMBC spectrum of ADP_S_-2 (**k**)

As shown in Fig. 5, the anomeric region of the ^1^H NMR spectrum showed at 5.2-5.5 ppm for ADP-1a, ADPs-1b, ADPs-2, ADPs-3a and ADPs-3b, indicating that five polysaccharides from *A. dahuricae Radix* were mainly composed of one type of sugars, which was α form. The chemical shifts from 3.3 to 4.5 ppm were assigned to the H-2 to H-6 protons.

The ^13^C NMR spectrum of ADP-1a and ADPs-2 had no signal at low field from 160 to 180 ppm, which illustrated it do not contain uronic acid. The ^13^C chemical shifts of ADP-1a and ADPs-1b was 99.51, 99.49 ppm (Fig. 5), which illustrated that it was an α-linked residue, and it was accordance with the analysis of ^1^H NMR. The corresponding hydrogen signal can be confirmed by the HMQC spectrum (Fig. 5) to be at 5.23, 5.24 ppm.

According to HMBC spectrum of ADP1a (Fig. 5), δ_H_ 5.23 showed correlations with the carbon signals at δ_C_ 76.6, 73.2 and 71.4, δ_H_ 3.7–3.8 showed correlations with the carbon signals at δc 76.6 and 71.4, δ_H_ 3.53–3.67 showed correlations with the carbon signals at δc 76.6, and 69.18, δ_H_ 3.2–3.5 showed correlations with the carbon signals at δ_C_ 99.5, 73.2, 71.4 and 60.2. The difference between ADP1a and ADP_S_-2 was δ_H_ 3.53–3.67 showed no correlations with the carbon signals.

### Scanning electron microscope analysis

**Fig. 6:**
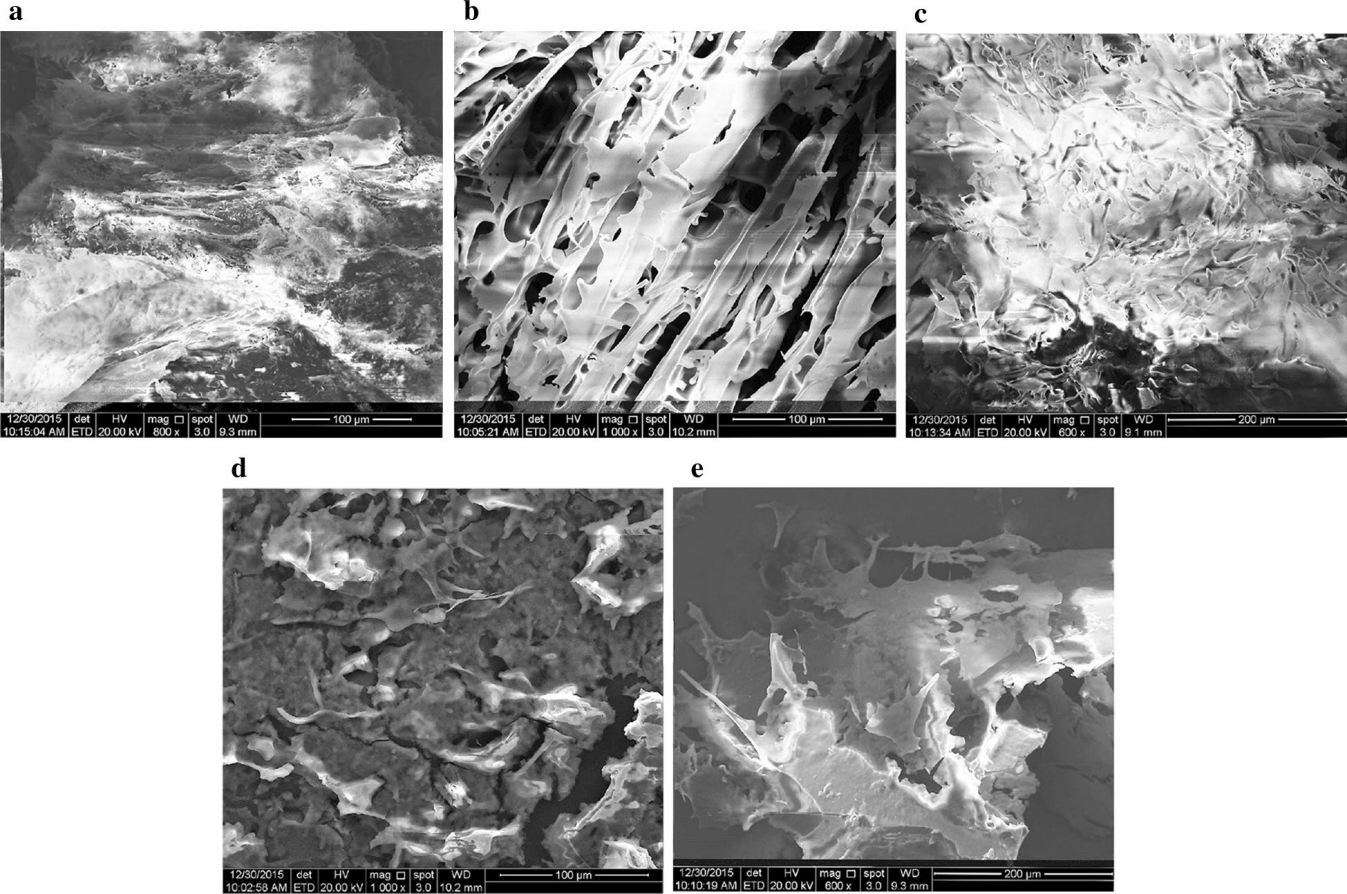
SEM of ADPs-1a (**a**), ADPs-1b (**b**), ADPs-2 (**c**), ADPs-3a (**d**) and ADPs-3b (**e**)

The SEM of ADPs-1a, ADPs-1b, ADPs-2, ADPs-3a and ADPs-3b were shown in Fig. 6. SEM images of ADPs-1a determined the surface was compact with close-packed arrays. There was multi-hole on the surface of ADPs-1b and was in flake accumulation. The surface of ADPs-2 was uneven in flocculent accumulation. The surface appearance of ADPs-3a was rough in flake accumulation. The surface topography of ADPs-3b was flat smooth in fragmental accumulation and the polysaccharide aggregate lined up tightly.

### Coagulation assays in vitro

Blood coagulation is a series of enzymatic processes, including the intrinsic pathway, extrinsic pathway and internal and external common pathway, finally fibrinogen is turned into fibrin, blood is turned from the sol into a gel state. Thrombin also plays an important role in the process of coagulation and blood coagulation. Therefore, PT is used to evaluate the coagulation factors V, VII and X in the overall efficiency of extrinsic clotting pathway. APTT is a test of the coagulation factors VIII, IX, XI, XII in the intrinsic clotting activity. TT is mainly a measure of transformation of fibrinogen to fibrin degree. FIB is employed to reflect the content of fibrinogen [14].

As shown in Table 2, compared with the blank group, ADPs-1b could significantly shorten PT and TT (*P* < 0.001) and could significantly increase the content of FIB (0.01 < *P* < 0.05), which indicated that ADPs-1b had procoagulant activities and exerted the procoagulant activities through intrinsic pathway, extrinsic pathway and increased the content of FIB. However, the activity of shortening PT exhibited a significant difference in relation to vitamin k_1_ (*P* < 0.001). ADPs-2 could significantly shorten APTT and PT (*P* < 0.001), and both of them had significant difference with the blank group (*P* < 0.001), and thus suggested that ADPs-2 had procoagulant activities and exerted the procoagulant activities through intrinsic pathway and extrinsic pathway. Compared with the blank group, ADPs-3a could significantly shorten APTT and TT (0.01 < *P* < 0.05, and 0.001 < *P* < 0.01, respectively) and could significantly increase the content of FIB (0.001 < *P* < 0.01), so the anticoagulant activities of ADPs-3a was associated with the intrinsic pathway and increased the content of FIB. Compared with the blank group, ADPs-3b could significantly shorten PT (*P* < 0.001), and could significantly increase the content of FIB (0.01 < *P* < 0.05), which indicated that ADPs-3b had procoagulant activities and exerted the activities through extrinsic pathway and increased the content of FIB.

**Table 2:** Effect of polysaccharides form *Angelicae dahuricae Radix* on plasma coagulation parameters

Group	Plasma coagulation parameters			
	APTT (s)	PT (s)	TT (s)	FIB (g/L)
Blank	15.23 ± 0.32	12.53 ± 0.34	15.00 ± 0.31	1.43 ± 0.04
Vitamin k_1_	14.33 ± 0.42***	12.08 ± 0.22*	13.28 ± 0.10***	1.85 ± 0.05**
ADPs-1a	15.27 ± 0.31	12.18 ± 0.17	14.65 ± 0.10	1.54 ± 0.15
ADPs-1b	15.30 ± 0.10	10.50 ± 0.26***^,&^	13.60 ± 0.24***	1.69 ± 0.07*
ADPs-2	11.80 ± 0.30***^,&^	11.08 ± 0.25***^,&^	14.50 ± 0.38	1.63 ± 0.20
ADPs-3a	14.73 ± 0.12*	12.48 ± 0.28	14.20 ± 0.47**	2.01 ± 0.11**
ADPs-3b	15.63 ± 0.25	11.30 ± 0.35***^,&^	14.60 ± 0.34	1.85 ± 0.11*

Data represent mean ± SD. *n* = 6

Compared with blank, *** *P* < 0.001, ** 0.001 <  *P* < 0.01,* 0.01 < *P* < 0.05

Compared with vitamin k_1_, ^&^ *P* < 0.001

According to reports in the literature [12], the water soluble part from *A. dahuricae Radix* can significantly shorten the clotting time of mice. In the present study, we demonstrated that ADPs-1b, ADPs-2, ADPs-3a and ADPs-3b were the components of procoagulant activity.

## Conclusions

Five polysaccharides were extracted and purified from *A. dahuricae Radix*. Coagulation assays in vitro indicated that ADPs-1b, ADPs-2, ADPs-3a and ADPs-3b had the procoagulant activity, The results imply that polysaccharides from *A. dahuricae Radix* has promising propects as hemostatics in medicines. However, Owing to the complex relationships existed between the structure and activities of the polysaccharides, further investigations in the structure and the relationship between the fine structure and procoagulant activity are required.

## Abbreviations

HPSEC: high performance gel permeation chromatography
GC: gas chromatography
FT-IR: Fourier transform infrared
NMR: spectrometer and nuclear magnetic resonance spectra
Mw: molecular weight
APTT: activated partial thromboplastin time
PT: prothrombin time
TT: thrombin time
FIB: fibrinogen
SD: standard deviation
ANOVA: analysis of variance
